# Convenient fabrication of conjugated polymer semiconductor nanotubes and their application in organic electronics

**DOI:** 10.1098/rsos.180868

**Published:** 2018-08-22

**Authors:** Lanchao Ma, Shuixing Dai, Xiaowei Zhan, Xinyang Liu, Yu Li

**Affiliations:** 1 College of Materials Science and Engineering, Beijing Key Laboratory of Special Elastomer Composite Materials, Beijing Institute of Petrochemical Technology, Beijing 102617, People's Republic of China; 2 Department of Materials Science and Engineering, College of Engineering, Peking University, Beijing 100871, People's Republic of China

**Keywords:** polymer nanotubes, core–shell structure, field-effect transistors, light-emitting nanostructures

## Abstract

Organic heterojunction is indispensable in organic electronic devices, such as organic solar cells, organic light-emitting diodes and so on. Fabrication of core–shell nanostructure provides a feasible and novel way to prepare organic heterojunction, which is beneficial for miniaturization and integration of organic electronic devices. Fabrication of nanotubes which constitute the core–shell structure in large quantity is the key for the realization of application. In this work, a simple and convenient method to prepare nanotubes using conjugated copolymer of perylene diimide and dithienothiophene (P(PDI-DTT)) was demonstrated. The relationship between preparation conditions (solvent atmosphere, solution concentration and pore diameter of templates) and morphology of nanostructure was studied systematically. P(PDI-DTT) nanotubes could be fabricated in regular shape and large quantity by preparing the solution with appropriate concentration and placing anodic aluminium oxide template with nanopore diameter of 200 nm in the solvent atmosphere. The tubular structure was confirmed by scanning electron microscopy. P(PDI-DTT) nanotubes exhibited electron mobility of 0.02 cm^2^ V^–1^ s^–1^ in field-effect transistors under ambient condition. Light-emitting nanostructures were successfully fabricated by incorporating tetraphenylethylene into polymer nanotubes.

## 1. Introduction

Study on organic semiconductors is in the ascendant, in virtue of diversity and easy tailoring of organic molecules [1–3]. Progressively, organic electronic devices based on organic semiconductors exhibited characteristics of light weight, low cost and flexibility [4], such as organic solar cells (OSCs) [5–7], organic light-emitting diodes (OLEDs) [8–10], organic light-emitting transistors (OLETs) [11–13] and so on. Indispensable component of the above devices is active layer which usually adopts organic semiconductor heterojunction that consists of at least two kinds of semiconductor. Organic semiconductor heterojunction played corresponding roles in different devices. In OSCs, different organic semiconductors that constitute heterojunction take part in the process of light absorption and exciton diffusion, and charge separation takes place at the interface of the two materials. In OLEDs and OLETs, certain materials transport charge carrier, while others give out light and the interfaces in the heterojunction have a great effect on the charge injection. Consequently, organic heterojunction is attracting more and more research interest [14–16].

Preparation of organic semiconductor heterojunction is the foundation of application and functionality for organic electronics. Bulk heterojunction is the most common which could be obtained by processing mixture of semiconductors in solution or co-evaporating semiconductors in vacuum, and therefore, the heterojunction exists in the whole structure. Layer-by-layer heterojunction could be obtained by depositing semiconductors one layer after another and the heterojunction takes up a small fraction of the whole architecture and the function of charge transport is performed by the respective layer [9,11]. Bulk heterojunction is generally adopted by OSCs [17], while layer-by-layer heterojunction is ordinarily used in OLEDs and OLETs [11,18]. Similarly, core–shell structure at nanometre scale provides a novel idea for construction of organic heterojunction, in which each component is continuous to ensure high charge carrier transport mobility. Moreover, nanostructure is propitious for integration and miniaturization of organic electronic devices.

Nanotubes could be used as either the core or the shell in the core–shell structure and some semiconductor nanotubes have been prepared [19–22]. Amphiphilic organic small molecules were the most used, and they can self-assemble to form nanotubes through supramolecular interaction [21–25]. An achiral π-conjugated fluorinated fused pyrazine derivative spread at the air/water interface can form nanotubes with lateral compression of 70 mN m^−1^ [20,26]. Poly[*N*-9′-heptadecanyl-2,7-carbazole-*alt*-5,5-(4′,7′-di-2-thienyl-2′,1′,3′-benzothiadiazole)] nanorods could be transformed from nanorods to nanotubes upon thermal annealing beyond melting point [27]. However, there were not many kinds of materials developed to prepare nanotubes, and the preparation methods were relatively homogeneous. Charge transport property was not investigated which is one of the crucially important characteristics of organic semiconductors.

**Figure 1.**
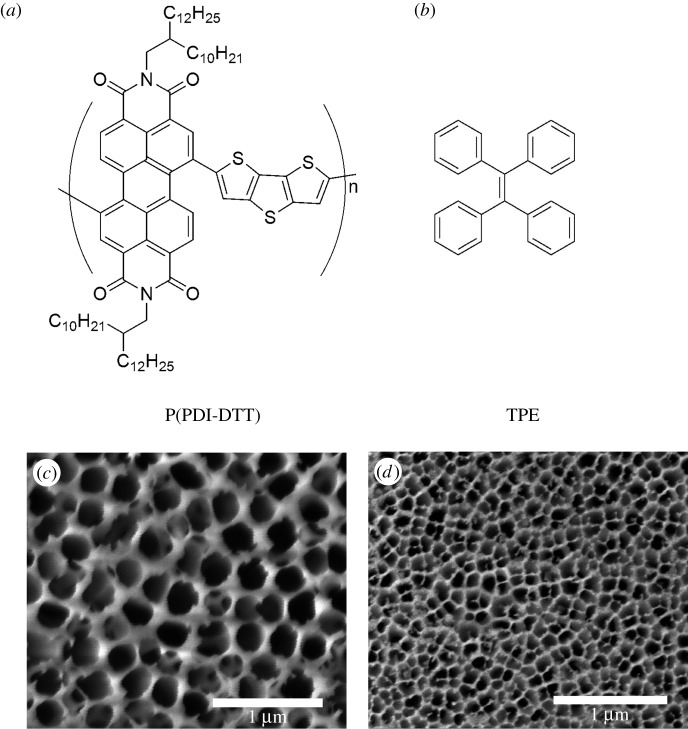
Chemical structure of P(PDI-DTT) (*a*) and TPE (*b*); right side (*c*) and reverse side (*d*) of AAO templates.

A conjugated copolymer of perylene diimide and dithienothiophene (P(PDI-DTT)) (structural formula of which is shown in figure 1*a*) is suitable for solution processing, and exhibits fine charge transport characteristic and excellent light absorption property [28]. It has been used as semiconductor layer in organic field-effect transistors (OFETs), the highest mobility of which reached 0.06 cm^2^ V^–1^ s^−1^ [29]. P(PDI-DTT) was also used as electron acceptor to prepare bulk p–n heterojunction in OSCs and the highest power conversion efficiency reached 3% [28,30]. P(PDI-DTT) showed a wide range of application in organic optoelectronics [31].

Fabrication of P(PDI-DTT) nanotubes to study their charge transport property and preparation of corresponding heterojunction are of great interest in terms of fundamental and application research. Wetting of anodic aluminium oxide (AAO) templates is a commonly used method to prepare one-dimensional nanomaterials [32]. Employment of polymer solution rather than polymer melt is more economic.

In this work, solution with moderate concentration, pore with appropriate diameter and structure and solvent atmosphere were selected as the preparation conditions. The solution flowed into nanopores favourably, and evaporated slowly. P(PDI-DTT) nanotubes of regular shape came into being and exhibited electron transport property in FET measurement. Blend solution of P(PDI-DTT) and tetraphenylethylene (TPE) (figure 1*b*) wetted AAO, giving nanostructures with P(PDI-DTT) as the shell and TPE as core.

## 2. Experimental methods

### 2.1. Materials

P(PDI-DTT) polymer was synthesized according to previously reported procedure [28]. TPE (98%) was purchased from Innochem and used directly without further purification. AAO templates were purchased from Whatman.

### 2.2. Preparation of polymer nanotubes

**Figure 2.**
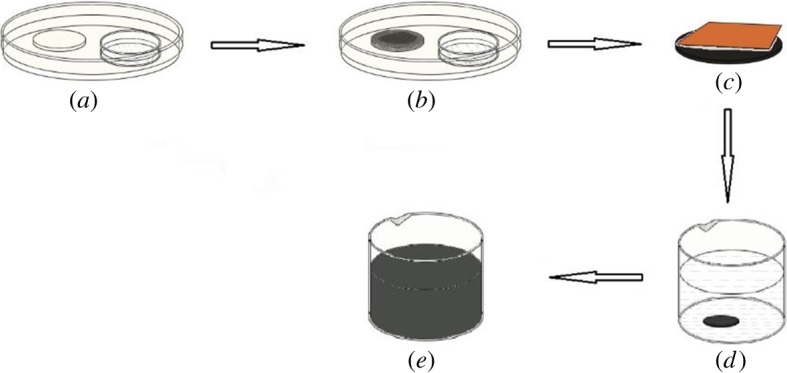
Process to prepare suspension of P(PDI-DTT) nanostructures.

Concentration of P(PDI-DTT) in chloroform was 5 mg ml^−1^. AAO templates were 60 µm in thickness, 2.5 cm in diameter and the aperture of the nanopore was 200 nm. AAO was made ready by ultrasonic cleaning in ethanol and acetone, respectively, then drying in an oven. One hundred microlitre solution of P(PDI-DTT) was dipped on the AAO template (figure 2*b*), which was placed in a Petri dish with chloroform atmosphere (figure 2*a*).

After 2 days, the template was taken out from the Petri dish. The polymer deposited on the AAO template was ground off by a sandpaper of 2000 mesh (figure 2*c*). The AAO template was got rid of by soaking into 3 M potassium hydroxide (KOH) aqueous solution for 15 min (figure 2*d*), then the polymer nanotubes were gathered in a beaker and rinsed with secondary water twice, and finally P(PDI-DTT) nanotubes were dispersed in ethanol (figure 2*e*).

### 2.3. Characterization of nanotubes

Optical microscopy (Olympus BX51) and scanning electron microscopy (SEM, Hitachi S-4800) were used to characterize the morphology of the nanotubes. The acceleration voltage of SEM was 15 kV. Transmission electron microscopy (TEM, JEM-1011) and corresponding selected area electron diffraction (SAED) were used to characterize the structure and crystallinity of the nanotubes on carbon-coated copper grids. The acceleration voltage of TEM was 100 kV. Inverted fluorescence microscope (Olympus IX83) was used to record fluorescence image of nanostructures. The fabrication of nanotube FET is described below [33]. The suspension was deposited on OTS-modified SiO**_2_** [34], which was then thermally annealed at 100°C in vacuum to remove solvent; subsequently, the drain and source electrodes were made by gold-layer sticking technique [35]. Measurement of charge carrier transport property was performed by Keithley 4200-SCS semiconductor characterization system. The mobility in the saturated regime was extracted from the following equation: *I*_DS_ *= C*_i_*μ*(*W/*2*L*)(*V*_GS_ *– V*_T_)^2^, where *I*_DS_ was the drain current, *C*_i_ the capacitance per unit area of the gate dielectric layer, and *V*_GS_ and *V*_T_ were the gate voltage and threshold voltage, respectively. Electrical characterization was performed in ambient atmosphere.

## 3. Results and discussion

### 3.1. Fabrication and morphology characterization of perylene diimide and dithienothiophene polymer nanotubes

Formation of nanostructure was greatly impacted by the preparation conditions, such as environmental atmosphere, pore diameter and structure and concentration of solution. Solvent atmosphere slowed solution evaporation, ensuring fluidity of the solution. In this work, the preparation process proceeded in a Petri dish (figure 2*a*), which consisted of a disc bottom and a cover made of glass. The container of chloroform in the Petri dish created solvent atmosphere, in virtue of the volatility of chloroform and impermeability of the Petri dish to some extent, which slowed down the evaporation of the P(PDI-DTT) solution. Consequently, P(PDI-DTT) in chloroform was more likely to flow into the pores, which was beneficial for nanostructure formation.

**Figure 3.**
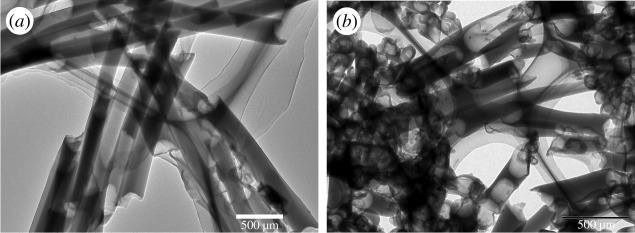
TEM images of P(PDI-DTT) nanotubes dispersed on carbon-coated copper grids. Preparation condition: (*a*) without container of chloroform in Petri dish; (*b*) with reverse side as the template.

Conversely, when the container of chloroform was not placed in the Petri dish in advance, the solution dropped on the AAO template evaporated rapidly to achieve vapour–liquid equilibrium, which led to discontinuity of nanostructure and loose packing in the nanotube (figure 3*a*).

The selection of appropriate side of AAO templates was crucial for the fabrication of nanotubes. The AAO template was commercially available, and the pore diameter value of 200 nm was marked on the external packing. Nevertheless, the fact was that the AAO template had two different sides: the nanopore diameter of the front side was about 240 nm and that of the reverse side was about 120 nm (figure 1*c,d*). The employment of the front side was beneficial for acquirement of nanotubes; however, the reverse side gave thinner rods (figure 3*b*), which implied that larger diameter of nanopores was helpful for the formation of nanotubes.

**Figure 4.**
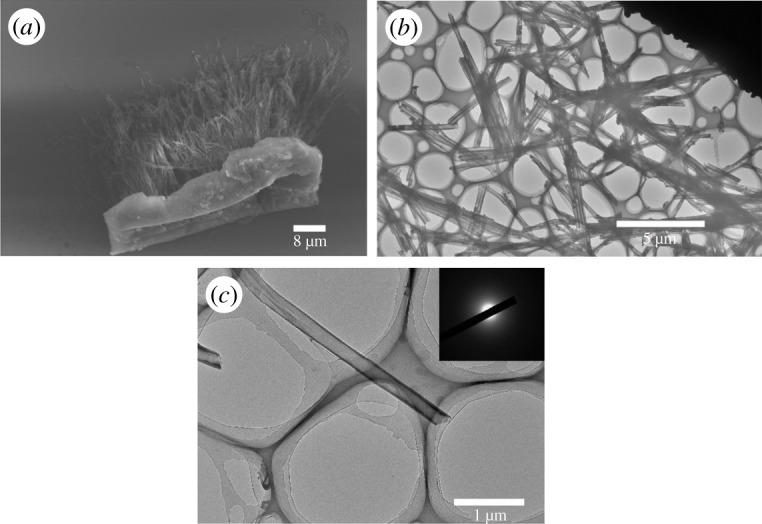
(*a*) SEM image of P(PDI-DTT) nanotubes in a bunch. (*b*) TEM image of P(PDI-DTT) nanotubes dispersed on carbon-coated copper grids. (*c*) TEM image of single P(PDI-DTT) nanotube.

The concentration of solution affected dispersity and integrality of nanotubes. Solution with high concentration (greater than 10 mg ml^−1^) led to much residue on the template, which was hard to grind off by sandpaper. After removing the template, the nanotubes conglutinated with each other by the residue, making it hard to disperse. Low concentration (less than 3 mg ml^−1^) fragmented nanotubes. The reason was that the solution could not soak the inwall continuously and completely or only formed thin layer on the wall of the pore, which was fragile. In such cases, the nanotubes were easy to be broken in the dispersion process. TEM (figure 4) showed that, for the nanotube, external diameter was 230 nm and the tube wall was thin. SAED revealed that the nanostructures were all amorphous. Consequently, appropriate concentration was necessary for nanotube fabrication. The formation of nanotubes was a dynamic equilibrium process to a great extent, and strict control of preparation condition was indispensable.

### 3.2. Field-effect transistor characterization of perylene diimide and dithienothiophene polymer nanotubes

**Figure 5.**
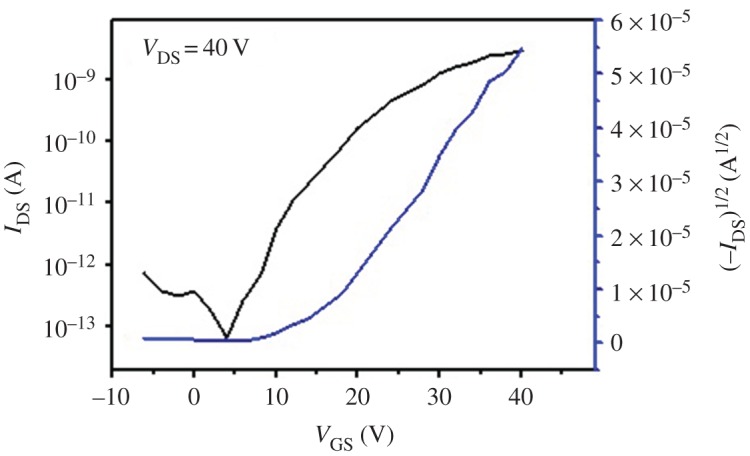
Transfer curve of polymer nanotube.

Ultimately, the charge carrier transport property was characterized by FET configuration under ambient atmosphere (figure 5). The P(PDI-DTT) nanotubes exhibited electron transport characteristics with mobility of 0.02 cm^2^ V^–1^ s^–1^, *I*_on_/*I*_off_ of 10^5^ and *V*_T_ of 10 V. The mobility was higher than that of film measured under inert atmosphere with the same bottom-gate top-contact configuration [28], but was lower than that of film measured with top-gate bottom-contact configuration [29] reported in previous work. The nanotubes exhibited environmental stability to a certain extent; however, it was still not excellent enough to obtain stable output curves. For the polymer of P(PDI-DTT), encapsulation was probably a feasible way to improve air stability.

### 3.3. Fabrication of core–shell structure

**Figure 6.**
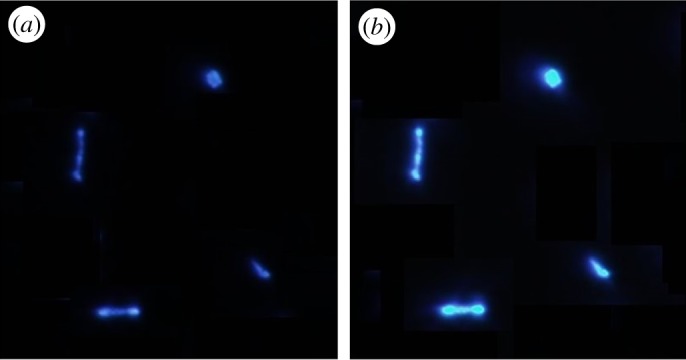
Fluorescence microscope image of the nanostructure with (*a*) low exciting intensity and (*b*) high exciting intensity.

As mentioned above, major application of nanotubes is the fabrication of heterojunction. TPE shows aggregate-induced emission. Polymer nanotubes incorporating TPE particles were obtained by wetting the AAO templates with mixed solution of P(PDI-DTT) and TPE in 1 : 3 weight ratio. Figure 6 shows inverted fluorescence microscope images of the nanostructure with exciting light of different intensity. It was clearly seen that TPE particles were capable of being encapsulated in the nanotubes and the particles that could not be encapsulated scattered on the cover glass. The light-emitting nanotubes were short, because TPE particles were irregular, cutting off the nanostructures when they were dispersed in ethanol. In this way, organic/polymer nanostructures with both moderate electron transport and light-emitting property were easily fabricated, which provided a new approach for preparing OLETs. The core–shell nanostructure made invisible nanostructure visible, and have potential application in cell imaging.

## 4. Conclusion

Nanotubes of P(PDI-DTT) in large quantity were successfully prepared. This work highlights the importance of preparation conditions. Solvent atmosphere, appropriate dimension of the pore and moderate solution concentration were beneficial for obtaining regular nanotubes. The nanotubes of P(PDI-DTT) exhibited preferable FET performance than film with the same device configuration. Electron mobility of P(PDI-DTT) nanotubes was 0.02 cm^2^ V^–1^ s^–1^, *I*_on_/*I*_off_ was 10^5^ and *V*_T_ was 10 V. It was also demonstrated that P(PDI-DTT) was able to encapsulate TPE particles, forming light-emitting nanostructures. In a word, P(PDI-DTT) nanotubes have potential application for future organic electronics owing to the hollow structure and electron transport property.

## Data accessibility

Preparation of P(PDI-DTT) and the fabrication of field-effect transistor based on nanotube was the key to reproduce the work. The detailed procedure to prepare P(PDI-DTT) was clearly elaborated in the supporting information of Zhan *et al*. [28] (‘A high-mobility electron-transport polymer with broad absorption and its use in field-effect transistors and all-polymer solar cells' (doi:10.1021/ja071760d)). Gold-layer sticking technique to fabricate field-effect transistor was clearly elaborated on page 1512 in [35] (‘High-performance air-stable bipolar field-effect transistors of organic single-crystalline ribbons with an air-gap dielectric’ (doi:10.1002/adma.200702145)). Data are available at Dryad (http://dx.doi.org/10.5061/dryad.2mg67hq) [36].

## References

[1] Wang J, Liu K, Ma L, Zhan X 2016 Triarylamine: versatile platform for organic, dye-sensitized, and perovskite solar cells. Chem. Rev. 116, 14 675–14 725. (10.1021/acs.chemrev.6b00432) 27960267

[2] Ball M et al. 2016 Macrocyclization in the design of organic n-type electronic materials. J. Am. Chem. Soc. 138, 12 861–12 867. (10.1021/jacs.6b05474) 27666433

[3] Osaka I, Takimiya K 2017 Naphthobischalcogenadiazole conjugated polymers: emerging materials for organic electronics. Adv. Mater. 29, 1605218 (10.1002/adma.201605218) 28240796

[4] Bao ZN 2016 Skin-inspired organic electronic materials and devices. MRS Bull. 41, 897–904. (10.1557/mrs.2016.247)

[5] Tang AL, Zhan CL, Yao JN, Zhou EJ 2017 Design of diketopyrrolopyrrole (DPP)-based small molecules for organic-solar-cell applications. Adv. Mater. 29, 1600013 (10.1002/adma.201600013) 27859743

[6] Cheng P, Zhan XW 2016 Stability of organic solar cells: challenges and strategies. Chem. Soc. Rev. 45, 2544–2582. (10.1039/C5CS00593K) 26890341

[7] Hutnan MPJ, Kaake LG 2016 Design principles for block polymer organic double heterojunction solar cells. Mater. Horiz. 3, 575–580. (10.1039/C6MH00293E)

[8] Higuchi T, Nakanotani H, Adachi C 2015 High-efficiency white organic light-emitting diodes based on a blue thermally activated delayed fluorescent emitter combined with green and red fluorescent emitters. Adv. Mater. 27, 2019–2023. (10.1002/adma.201404967) 25664428

[9] Perumal A et al 2015 High-efficiency, solution-processed, multilayer phosphorescent organic light-emitting diodes with a copper thiocyanate hole-injection/hole-transport layer. Adv. Mater. 27, 93–100. (10.1002/adma.201403914) 25382072 PMC4315901

[10] Matsuoka K, Albrecht K, Yamamoto K, Fujita K 2017 Mulifunctional dendritic emitter: aggregation-induced emission enhanced, thermally activated delayed fluorescent material for solution-processed multilayered organic light-emitting diodes. Sci. Rep. 7, 41780 (10.1038/srep41780) 28139768 PMC5282576

[11] Capelli R, Toffanin S, Generali G, Usta H, Facchetti A, Muccini M 2010 Organic light-emitting transistors with an efficiency that outperforms the equivalent light-emitting diodes. Nat. Mater. 9, 496–503. (10.1038/nmat2751) 20436466

[12] Zhang CC, Chen PL, Hu WP 2016 Organic light-emitting transistors: materials, device configurations, and operations. Small 12, 1252–1294. (10.1002/smll.201502546) 26833896

[13] Soldano C et al. 2014 ITO-free organic light-emitting transistors with graphene gate electrode. ACS Photonics 1, 1082–1088. (10.1021/ph500289s)

[14] Menke SM et al. 2016 Limits for recombination in a low energy loss organic heterojunction. ACS Nano 10, 10 736–10 744. (10.1021/acsnano.6b06211) 27809478

[15] Wu J, Li Q, Xue G, Chen H, Li H 2017 Preparation of single-crystalline heterojunctions for organic electronics. Adv. Mater. 29, 1606101 (10.1002/adma.201606101) 28234418

[16] Street RA 2016 Electronic structure and properties of organic bulk-heterojunction interfaces. Adv. Mater. 28, 3814–3830. (10.1002/adma.201503162) 26603977

[17] Cheng P, Li G, Zhan X, Yang Y 2018 Next-generation organic photovoltaics based on non-fullerene acceptors. Nat. Photonics 12, 131–142. (10.1038/s41566-018-0104-9)

[18] Tang X et al. 2018 Efficient nondoped blue fluorescent organic light-emitting diodes (OLEDs) with a high external quantum efficiency of 9.4% @ 1000 cd m^−2^ based on phenanthroimidazole–anthracene derivative. Adv. Funct. Mater. 28, 1705831 (10.1002/adfm.201705813)

[19] Hu X, Chen G, Wang X, Wang H 2015 Tuning thermoelectric performance by nanostructure evolution of a conducting polymer. J. Mater. Chem. A 3, 20 896–20 902. (10.1039/C5TA07381B)

[20] Yao PP, Wang HF, Chen PL, Zhan XW, Kuang X, Zhu DB, Liu MH 2009 Hierarchical assembly of an achiral π-conjugated molecule into a chiral nanotube through the air/water interface. Langmuir 25, 6633–6636. (10.1021/la901435s) 19459675

[21] Wang Y, Huang Z, Kim Y, He Y, Lee M 2014 Guest-driven inflation of self-assembled nanofibers through hollow channel formation. J. Am. Chem. Soc. 136, 16 152–16 155. (10.1021/ja510182x) 25372152

[22] Ma XJ, Zhang YB, Zhang YF, Peng C, Che YK, Zhao JC 2015 Stepwise formation of photoconductive nanotubes through a new top-down method. Adv. Mater. 27, 7746–7751. (10.1002/adma.201503771) 26485110

[23] Shao H, Seifert J, Romano NC, Gao M, Helmus JJ, Jaroniec CP, Modarelli DA, Parquette JR 2010 Amphiphilic self-assembly of an n-type nanotube. Angew. Chem. Int. Ed. 49, 7688–7691. (10.1002/anie.201003415) 20821782

[24] Sun Y, He C, Sun K, Li Y, Dong HL, Wang ZH, Li ZB 2011 Fine-tuned nanostructures assembled from l-lysine-functionalized perylene bisimides. Langmuir 27, 11 364–11 371. (10.1021/la202107r) 21823641

[25] Shao H, Gao M, Kim SH, Jaroniec CP, Parquette JR 2011 Aqueous self-assembly of L-lysine-based amphiphiles into 1D n-type nanotubes. Chem. Eur. J. 17, 12 882–12 885. (10.1002/chem.201102616) 22167876

[26] Wang HF et al. 2009 Fused-ring pyrazine derivatives for n-type field-effect transistors. ACS Appl. Mater. Interfaces 1, 1122–1129. (10.1021/am900093p) 20355900

[27] Aziz F, Bakar NA, Bashir S, Alhummiany H, Bawazeer T, Alsenany N, Mahmoud A, Supangat A, Sulaiman K 2017 Effective transformation of PCDTBT nanorods into nanotubes by polymer melts wetting approach. J. Saudi Chem. Soc. 21, 720–730. (10.1016/j.jscs.2017.03.005)

[28] Zhan XW et al. 2007 A high-mobility electron-transport polymer with broad absorption and its use in field-effect transistors and all-polymer solar cells J. Am. Chem. Soc. 129, 7246–7247. (10.1021/ja071760d) 17508752

[29] Zhang L et al. 2010 Top-gate organic thin-film transistors constructed by a general lamination approach. Adv. Mater. 22, 3537–3541. (10.1002/adma.201000123) 20648517

[30] Cheng P, Zhao X, Zhou W, Hou J, Li Y, Zhan X 2014 Towards high-efficiency non-fullerene organic solar cells: matching small molecule/polymer donor/acceptor. Org. Electron. 15, 2270–2276. (10.1016/j.orgel.2014.06.025)

[31] Yan CQ, Barlow S, Wang ZH, Yan H, Jen AK Y, Marder SR, Zhan XW 2018 Non-fullerene acceptors for organic solar cells. Nat. Rev. Mater. 3, 18003 (10.1038/natrevmats.2018.3)

[32] Steinhart M, Wendorff JH, Greiner A, Wehrspohn RB, Nielsch K, Schilling J, Choi J, Gösele U 2002 Polymer nanotubes by wetting of ordered porous templates. Science 296, 1997 (10.1126/science.1071210) 12065828

[33] Liu Y, Wang HF, Dong HL, Jiang L, Hu WP, Zhan XW 2013 High performance photoswitches based on flexible and amorphous D-A polymer nanowires. Small 9, 294–299. (10.1002/smll.201201332) 22987536

[34] Wen YG et al. 2009 Improvements in stability and performance of *N*,*N*′-dialkyl perylene diimide-based n-type thin-film transistors. Adv. Mater. 21, 1631–1635. (10.1002/adma.200802934)

[35] Tang Q, Tong Y, Li H, Ji Z, Li L, Hu W, Liu Y, Zhu D 2008 High-performance air-stable bipolar field-effect transistors of organic single-crystalline ribbons with an air-gap dielectric Adv. Mater. 20, 1511–1515. (10.1002/adma.200702145)

[36] Ma L, Dai S, Zhan X, Liu X, Li Y 2018 Data from: Convenient fabrication of conjugated polymer semiconductor nanotubes and their application in organic electronics *Dryad Digital Repository*. (10.5061/dryad.2mg67hq) PMC6124030 30225076

